# Suicide Prevention Strategies for Older People with Mental Health Challenges in Tower Hamlets Centre for Mental Health: A Qualitative Study

**DOI:** 10.1192/bjo.2025.10450

**Published:** 2025-06-20

**Authors:** Huan Tan, Tatjana Fawzi, Sonal Shah, Fergus Gordon, Hugh Grant-Peterkin

**Affiliations:** East London NHS Foundation Trust, London, United Kingdom

## Abstract

**Aims:** Suicide remains one of the leading causes of death worldwide, with rates among older adults increasing steadily. Older people face higher suicide completion rates, especially in 45–49 and 90+ age group. The impact of suicide on society is profound, underlining the need for targeted interventions for this demographic.

This project follows the Triple Aim framework to improve overall health system by:

Enhancing suicide prevention in older populations through mental well-being promotion.

Increasing awareness and improving patients’ experience by creating a supportive, responsive environment.

Developing a replicable model across healthcare settings, contributing to broader suicide prevention efforts.

**Methods:** Focus groups were conducted with 3 cohorts: patients, families and staff. 4–8 participants were recruited for each group using purposive sampling method. Semi-structured interview was conducted to explore their views on suicide prevention, their challenges, and expectations.

**Results:** Many patients identified negative emotions: stress and overthinking are contributing factors. Many find feelings of guilt/hopelessness, bereavement particularly challenging. Additionally, social isolation, physical health problems and poor sleep also lead to suicide.

According to staff, many patients lack access to service due to language barrier, immobility/disabilities, socio-economic deprivation and limited access to technologies. Cultural beliefs and stigma play a major role. Staff also highlighted that role transition to retirement results loneliness/isolation.

Preventive strategies include normalisation and promoting awareness in public. Having representation in peer support group can improve stigma in minority. Social interventions can aid role transition and provide sense of belonging. Integrated care with multiple touchpoints from emergency care to community/GP follow-ups alongside with multidisciplinary approach with occupational therapist and psychologist are crucial in providing patient-centred care.

**Conclusion:** These focus groups underscore the importance of suicide prevention for older people. The insight gained will inform future strategies and prioritise change ideas in our service.

